# Once-weekly semaglutide administered after laparoscopic sleeve gastrectomy: Effects on body weight, glycemic control, and measured nutritional metrics in Japanese patients having both obesity and type 2 diabetes

**DOI:** 10.1016/j.obpill.2023.100098

**Published:** 2024-01-03

**Authors:** Rieko Kanai, Sachiho Kinoshita, Izumi Kanbe, Mariko Sameda, Shuhei Yamaoka, Osamu Horikawa, Yasuhiro Watanabe, Ichiro Tatsuno, Kohji Shirai, Takashi Oshiro, Atsuhito Saiki

**Affiliations:** a Department of Medical Nutrition, Toho University Sakura Medical Center, Sakura, Japan; b Center of Diabetes, Endocrine and Metabolism, Toho University Sakura Medical Center, Sakura, Japan; c Chiba Prefectural University of Health Sciences, Chiba, Japan; d Department of Internal Medicine, Mihama Hospital, Chiba, Japan; e Department of Surgery, Toho University Sakura Medical Center, Sakura, Japan

**Keywords:** Once-weekly glucagon-like peptide 1 receptor agonist, Semaglutide, Laparoscopic sleeve gastrectomy, Type 2 diabetes, Formula diet

## Abstract

**Background:**

Glucagon-like peptide (GLP)-1 analogue may be useful for controlling weight recurrence and diabetes relapse after bariatric surgery, but may also adversely affect the measured nutritional metrics. This study aimed to investigate the effect of treatment with once-weekly semaglutide after laparoscopic sleeve gastrectomy (LSG) in patients with type 2 diabetes (T2D). We also examined the effects of combined use with a low-energy, high-protein formula diet (FD).

**Methods:**

This study was a single-center retrospective database analysis. We enrolled 29 Japanese patients with T2D who underwent LSG, and more than 12 months later received semaglutide. The patients were divided retrospectively into a FD group (=6) and a conventional diet (CD) group (n = 23).

**Results:**

BMI and HbA1c decreased significantly by 10.7 kg/m^2^ and 1.1 %, respectively, 12 months after LSG, and decreased by an additional 1.6 kg/m^2^ and 0.6 % after 12-months of treatment with semaglutide. Decreases in serum albumin, vitamin B12 and zinc were observed only after semaglutide administration. A ratio of energy from protein, fat and carbohydrates changed from 13:31:56 before to 19:30:50 after LSG, and from 17:32:51 before to 15:29:56 after semaglutide. Skeletal muscle ratio, which is the ratio of skeletal muscle mass to body weight, increased after LSG, but did not change after semaglutide. FD group showed a significant increase in skeletal muscle mass per 1 % body weight compared to CD group during semaglutide treatment.

**Conclusion:**

Semaglutide after LSG in patients with obesity and T2D resulted in additional weight reduction and improved glycemic control, but worsened measured nutritional metrics. Administration of a low-energy, high protein formula diet may ameliorate adverse nutritional effects of semaglutide in patients with T2D after LSG. (Ethics Committee of Toho University Sakura Medical Center approval number S18061)

## Graphical abstract

**Figure:**
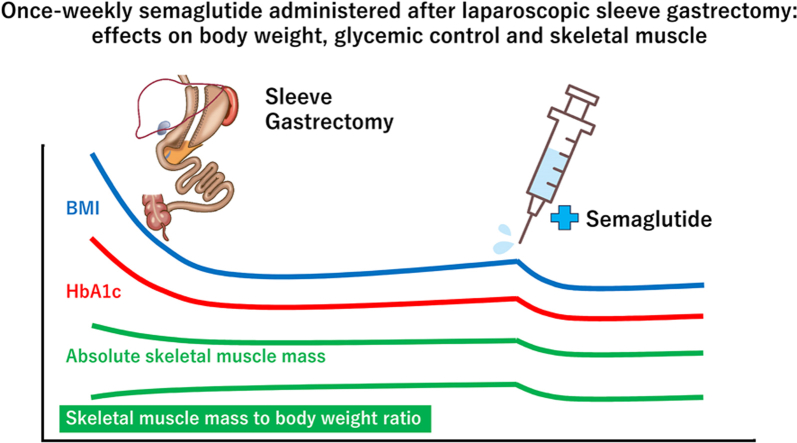

## 1 Introduction

Obesity is associated with an increased risk of obesity-related comorbidities including type 2 diabetes mellitus, dyslipidemia, hypertension, and sleep apnea syndrome [1,2]. However, conventional approaches such as lifestyle modification, dietary control and increasing physical activity are usually insufficient to achieve satisfactory weight reduction in patients with obesity [3,4]. Bariatric surgery has been demonstrated to be the most effective weight reduction therapy available. Laparoscopic sleeve gastrectomy (LSG) as a single-stage procedure has gained popularity worldwide [5]. LSG has become a popular treatment option also in Japan. A nationwide survey conducted by the retrospective study group “Japanese Survey of Morbid and Treatment-Resistant Obesity” (J-SMART) reported percent total weight loss (TWL) of 29.9 % and complete diabetes remission rate of 75.6 % at 2 years after LSG [6]. On the other hand, 26.8 % of subjects had recurrence of diabetes along with suboptimal weight reduction and excess weight recurrence within 5 years after LSG in the J-SMART study [7]. In several studies, 16–37 % of subjects showed significant weight recurrence in the long term [8,9]. An effective standard therapy to control weight recurrence after bariatric surgery has not been established.

Trials evaluating a glucagon-like peptide 1 (GLP-1) analogue have shown improved cardiovascular outcomes in patients with obesity without diabetes as well as with type 2 diabetes who were at high risk for cardiovascular events [[10], [11], [12]]. Semaglutide is a GLP-1 analogue that binds to the GLP-1 receptor in pancreatic β-cells to induce insulin secretion in a glucose concentration-dependent manner [12]. Once weekly semaglutide is used worldwide as a treatment for type 2 diabetes with obesity [13]. In the SUSTAIN-6 clinical trial, the semaglutide 1.0 mg group showed a significant reduction in HbA1c and a mean weight of 4.3 kg lower compared to the placebo group [12]. Furthermore, a single center retrospective observational study showed that semaglutide had a safe weight reduction effect in patients experiencing weight recurrence after bariatric surgery [14].

Bariatric surgery may lead to deficiency of several nutritional elements, including vitamins and minerals, of which protein deficiencies are particularly important [15]. It is critical to screen for nutritional deficiencies prior to bariatric surgery and at regular intervals after bariatric surgery, and encourage adherence to nutritional supplementation [15]. A formula diet (FD) containing high protein, low carbohydrate, low fat and sufficient vitamins and minerals is effective in reducing body weight [16] and maintaining or increasing skeletal muscle mass [17]. FD is recommended before bariatric surgery as a very low calorie diet regimen aiming to improve perioperative outcomes [18,19]. Postoperative FD also may be effective to prevent protein deficiency and skeletal muscle mass loss after LSG [6]. On the other hand, semaglutide has adverse effects such as delayed gastric emptying and appetite loss [20], which may affect nutritional intake and body composition. Administration of semaglutide to patients after bariatric surgery may affect their measured nutritional metrics. We hypothesized that semaglutide might be effective against weight recurrence and diabetes relapse after LSG, but might worsen measured nutritional metrics and skeletal muscle indices.

The aim of this study was to investigate the effect of once-weekly semaglutide given after LSG on postoperative weight and glycemic control, as well as on measured nutritional metrics and body composition in Japanese patients with type 2 diabetes. In addition, the effects of FD on these indices were also examined.

## 2 Methods

This study was a single-center retrospective database analysis. Inclusion criteria were Japanese patients with type 2 diabetes treated with LSG at Toho University Medical Center from January 2010 to December 2021 (181 patients) who started once-weekly semaglutide (Ozempic®), which was launched in June 2020, at least one year after LSG. Subjects with end-stage renal disease, congestive heart failure, coronary heart disease, decompensated cirrhosis, acute/chronic inflammation, malignancy and pregnancy were excluded. A total of 29 patients (17 males and 12 females with an average age of 47.2 years) were evaluated in the study. LSG is the only bariatric procedure which is covered by the national health insurance in Japan, and all patients in this study received medical care under this system. The criterion for surgical indication was body mass index (BMI) higher than 35 kg/m^2^ with type 2 diabetes, dyslipidemia, hypertension and/or sleep apnea syndrome. Patients with weight recurrence (5 % weight recurrence from nadir) or worsening glycaemic control (HbA1c ≥ 6.5 %) were eligible for treatment with semaglutide, however, this was determined on a case-by-case basis. Patients with adverse effects such as gastrointestinal symptoms were excluded. Nineteen patients received once-weekly semaglutide as the first GLP-1 analogue therapy, while 10 patients switched from another GLP-1 analogue to once-weekly semaglutide (dulaglutide 0.75 mg: 5 cases, liraglutide: 4 cases [1.8 mg: 1 case, 1.2 mg: 1 case, 0.9 mg: 2 cases], exenatide 2 mg/week: 1 case). The national health insurance in Japan allows escalation of the semaglutide dosage to 1.0 mg once a week. The final weekly dose was 0.25 mg in 2 patients, 0.5 mg in 4 patients, and 1.0 mg in 23 patients. The reasons why the final dose of semaglutide was not the maximum dose of 1.0 mg were as follows. 4 cases of adequate efficacy, 1 case of nausea, and 1 case of economic reasons. Semaglutide was initiated a median of 52 (22–77) months after LSG. The following preoperative and postoperative data were collected from medical records: age, anthropometric measurements, visceral fat area (VFA), subcutaneous fat area (SFA), skeletal muscle mass, blood pressure, glycated hemoglobin (HbA1c), fasting blood glucose, fasting serum *C*-peptide (CPR), lipid markers, liver function, renal function, vitamins/minerals, and apnea-hypopnea index. VFA was determined using computed tomography (CT). The CT scan was performed at the umbilical level with the subject resting in supine position. SFA was calculated by subtracting VFA from total fat area. Skeletal muscle mass was measured by bioelectrical impedance analysis using InBody 720 (InBody Japan Co., Ltd., Tokyo, Japan) [21]. Information of nutrition was also collected. Calorie intake and dietary composition were assessed every 2 weeks by standardized interviews conducted by trained dieticians using a computerized database and analysis of semi-quantitative food records of 3 consecutive days.

Each parameter was evaluated at 12 months after LSG and after 12 months of once-weekly semaglutide treatment (“after 12 months semaglutide” hereinafter). The primary endpoints were change in HbA1c and BMI. The secondary endpoints were the change in skeletal muscle ratio, which is the ratio (%) of skeletal muscle mass (kg) to total body weight (kg), and various measured nutritional metrics. For the evaluation after 12 months semaglutide, the patients were divided retrospectively into two groups: a group using formula diet (FD group; n = 6) and a group on conventional diet (CD group; n = 23). In both groups, patients were instructed to aim to consume a total daily calorie intake of 20 kcal/kg/day and to consume at least 60 g/day of protein. In the FD group, one meal per day was replaced with a formula diet; MICRODIET (Sunny Health Co. Ltd., Japan) or ObeCure (US Cure Co. Ltd., Japan). One serving of FD contains 22.0 g of protein, 2.0 g of fat, 15.0 g of carbohydrate as macronutrients, and 0.9 mg of vitamin B1, 2.2 μg of vitamin B12, 163 μg of folic acid, 4.2 μg of vitamin D, 10.1 mg of iron and 5.0 mg of zinc as micronutrients. All participants in the CD group or FD group were prescribed daily supplements of multivitamins and multiminerals (Nature Made; Otsuka Pharmaceutical Co., Ltd., Japan), which contains 1.5 mg of vitamin B1, 3.0 μg of vitamin B12, 240 μg of folic acid, 5.0 μg of vitamin D, 4.0 mg of iron and 6.0 mg of zinc. Micronutrient intakes were based on the Dietary Reference Intakes for Japanese (Health Service Bureau, Ministry of Health, Labour and Welfare, JAPAN) and any nutrient deficiencies were addressed individually with supplements.

The results are expressed as mean ± standard deviation or median (interquartile range), or percentage. SPSS 15.0 (SPSS Inc., Chicago, Ill, USA) was used in all statistical analyses. For two-group comparisons, parametric data were analyzed using Student's *t*-test and non-parametric data were analyzed using Mann-Whitney *U* test. Wilcoxon signed-rank test was used for comparisons before and after 12 months of LSG and semaglutide administration, respectively. Fisher's exact test was used for the ratio of energy from protein, fat and carbohydrates (PFC ratio). A two-sided p value of 0.05 was considered statistically significant.

## 3 Results

### 3.1 Background characteristics and metabolic parameters at 12 months after LSG and after 12 months of semaglutide treatment

**Fig. 1:**
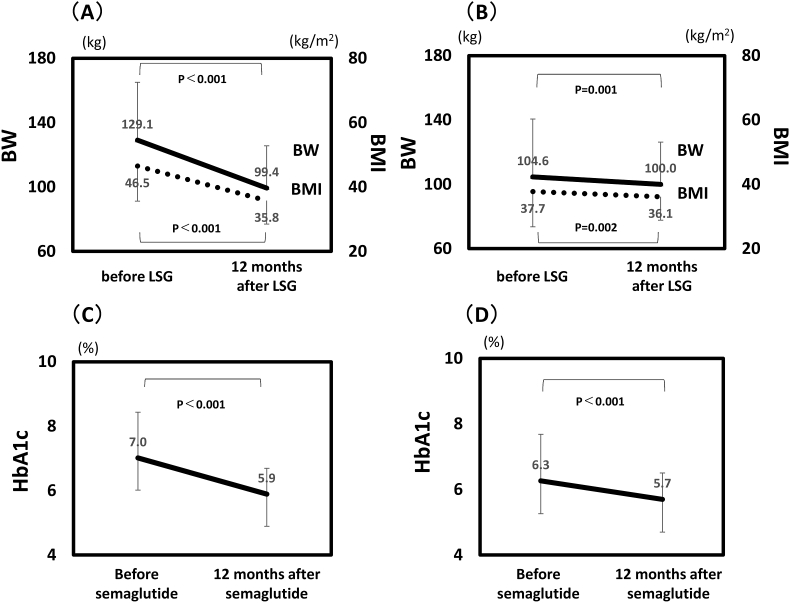
Change in BW, BMI and HbA1c before and after LSG and semaglutide treatment. BW, BMI (A) (B), and HbA1c (C) (D). Parameters were measured before LSG, 12 months after LSG (A) (C), before semaglutide, and after 12-month semaglutide (B) (D). Semaglutide was initiated a median of 52 (22–77) months after LSG. Data are presented as mean ± SD. BW, body weight; BMI, body mass index.

**Fig. 2:**
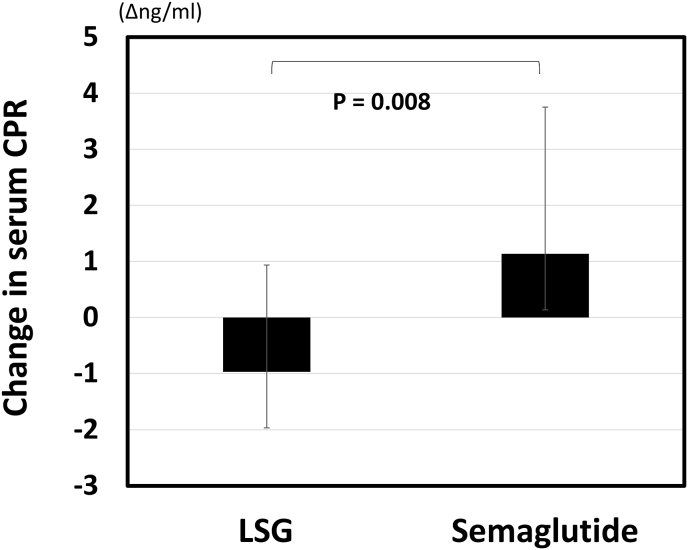
Comparison of changes in serum CPR at 12 months after LSG and after 12 months of once-weekly semaglutide treatment. Data are presented as mean ± SD. CPR, C peptide immunoreactivity; LSG, laparoscopic sleeve gastrectomy.

At the initial visit, the mean body weight, BMI and HbA1c of all subjects were 129.1 kg, 46.5 kg/m^2^ and 7.0 %, respectively. Other background parameters are shown in Table 1. First, changes in anthropometric and metabolic parameters during 12 months after LSG were compared with those during 12 months of semaglutide treatment (Fig. 1A and B, Table 2). From before LSG to 12 months after LSG, mean body weight decreased significantly from 129.1 to 99.4 kg (−29.7 kg), mean BMI decreased from 46.5 to 35.8 kg/m^2^ (−10.7 kg/m^2^), mean VFA decreased from 237 to 122 cm^2^ (−115 cm^2^), and mean HbA1c decreased from 7.0 to 5.9 ​% (−1.1 ​%). The absolute changes in skeletal muscle mass decreased from 35.7 to 31.9 ​kg (−10.6 ​%), while the mean skeletal muscle ratio increased from 28.4 to 33.2 ​% (+4.8 ​%) after LSG. In addition, SFA, body fat percentage, fasting glucose, CPR decreased significantly (Fig. 2). Thereafter, body weight (p = 0.042), BMI (p = 0.022), VFA (p < 0.001), and HbA1c (p = 0.016) increased significantly until once-weekly semaglutide treatment was initiated. From before starting semaglutide to after 12 months of treatment, mean body weight decreased from 104.6 to 100.0 kg (−4.6 kg), mean BMI decreased from 37.7 to 36.1 kg/m^2^ (−1.6 kg/m^2^), mean VFA decreased from 200 to 153 cm^2^ (−47 cm^2^), and mean HbA1c decreased from 6.3 to 5.7 % (−0.6 %). CPR tended to increase (Fig. 2). The skeletal muscle ratio did not change after 12 months of semaglutide treatment. The decreases in body weight, BMI, body fat percentage, HbA1c, CPR, as well as the increases in skeletal muscle ratio was greater after LSG than after semaglutide treatment.

**Table 1:** Background characteristics.

	Before LSG
Age (Y)	47.2 ± 8.7
Gender (male/female)	17/12
Height (cm)	166.2 ± 9.3
BW (kg)	129.1 ± 35.9
BMI (kg/m^2^)	46.5 ± 11.0
VFA (cm^2^)	210.3 (166.2–292.0)
SFA (cm^2^)	495.9 (425.3–666.7)
Skeletal muscle ratio (%)	28.0 (26.3–29.9)
Systolic BP (mm Hg)	137.3 ± 13.0
Diastolic BP (mm Hg)	81.1 ± 8.3
Fasting glucose (mg/dl)	112.5 (101.3–134.0)
HbA1c (%)	7.0 ± 1.4
CPR (ng/ml)	3.4 (2.5–4.1)
TC (mg/dL)	179.3 ± 41.3
TG (mg/dL)	155.0 (106.5–189.3)
HDL-C (mg/dL)	39.4 ± 8.6
AST (IU/L)	33.5 (26.3–40.8)
ALT (IU/L)	43.5 (26.5–54.3)
Cr (mg/dL)	0.8 (0.6–0.9)
Urinary albumin (mg/gCr)	16.9 (5.1–51.3)
Uric acid (mg/dL)	6.5 (5.7–7.7)
Hb (g/dl)	14.2 ± 1.9
Alb (g/dl)	4.2 ± 0.4
ChE (IU/L)	379.0 ± 82.6
Vitamin B1 (μg/dl)	4.8 (4.3–5.7)
Vitamin B12 (pg/dl)	447.0 (364.8–559.0)
Folic Acid (ng/ml)	9.7 (5.9–13.4)
Vitamin D (ng/ml)	13.6 (11.1–16.5)
Zinc (μg/dl)	71.5 (64.3–77.5)
Iron (mg/dl)	82.5 (68.5–109.5)

Data are presented as mean ± SD or median (interquartile range). BW, body weight; BMI, body mass index; VFA, Visceral fat area; SFA, subcutaneous fat area; FBG, fasting blood glucose; HbA1c, glycosylated hemoglobin; CPR, C peptide immunoreactivity; TC, total cholesterol; TG, triglyceride; HDL-C, high-density lipoprotein cholesterol; AST, aspartate aminotransferase; ALT, alanine aminotransferase; Cr, creatinine; Hb, hemoglobin; Alb, albumin; ChE, cholinesterase.

**Table 2:** Background characteristics and comparison of anthropometric and nutritional status at 12 months after LSG and after 12 months of semaglutide treatment.

	Before LSG	12 months after LSG	p-value	Before semaglutide	After 12 months semaglutide	p-value	p-value LSG vs semaglutide
VFA (cm^2^)	210.3 (166.2–292.0)	124.6 (93.3–194.1)	<0.001	210.9 (108.2–262.8)	133.6 (86.4–209.8)	0.011	0.034
SFA (cm^2^)	495.9 (425.3–666.7)	373.8 (328.4–485.9)	0.001	386.8 (319.2–636.4)	359.0 (246.6–523.5)	0.010	0.128
Skeletal muscle ratio (%)	28.0 (26.3–29.9)	31.2 (29.1–36.6)	<0.001	30.5 (26.8–34.9)	30.7 (28.0–34.9)	0.538	<0.001
Hb (g/dl)	14.2 ± 1.9	14.3 ± 1.8	0.747	14.5 ± 1.9	14.5 ± 1.7	1.000	0.763
Alb (g/dl)	4.2 ± 0.4	4.3 ± 0.4	0.164	4.1 ± 0.4	4.0 ± 0.4	0.082	0.048
ChE (IU/L)	379.0 ± 82.6	344.4 ± 73.7	0.025	355.6 ± 81.1	334.7 ± 80.1	0.041	0.973
Vitamin B1 (μg/dl)	4.8 (4.3–5.7)	5.0 (4.3–5.4)	0.877	4.6 (3.9–5.1)	4.3 (3.7–5.0)	0.222	0.586
Vitamin B12 (pg/dl)	447.0 (364.8–559.0)	454.0 (368.0–568.5)	0.520	522.0 (388.5–631.0)	399.0 (343.0–589.3)	0.007	0.039
Folic Acid (ng/ml)	9.7 (5.9–13.4)	8.1 (5.7–13.5)	0.488	8.1 (5.8–11.9)	7.6 (5.4–14.8)	0.550	0.375
Vitamin D (ng/ml)	13.6 (11.1–16.5)	25.2 (12.2–30.8)	0.039	23.2 (17.6–36.4)	27.7 (17.6–39.3)	0.279	0.076
Zinc (μg/dl)	71.5 (64.3–77.5)	72.0 (62.3–80.0)	0.849	78.0 (64.3–85.0)	69.5 (59.5–79.0)	0.023	0.100
Iron (mg/dl)	82.5 (68.5–109.5)	98.0 (70.3–117.0)	0.346	81.5 (61.0–94.3)	81.5 (66.3–100.5)	0.273	0.940

Data are presented as mean ± SD or median (interquartile range). VFA, Visceral fat area; SFA, subcutaneous fat area; Hb, hemoglobin; Alb, albumin; ChE, cholinesterase; LSG, laparoscopic sleeve gastrectomy.

### 3.2 Changes in measured nutritional metrics at 12 months after LSG and after 12 months of semaglutide treatment

First, changes in serum nutritional parameters during 12 months after LSG were analyzed (Table 2). Mean vitamin D increased significantly from 13.6 to 25.2 ​ng/ml (+11.2 ​ng/ml) and other serum nutritional parameters did not change from baseline to 12 months after LSG. On the other hand, from before starting semaglutide to after 12 months of treatment, mean vitamin B12 decreased significantly from 567 to 494 ​μg/dl (−73㎍/dl), zinc decreased significantly from 76.4 to 70.6 ​μg/dl (−5.8 ​μg/dl), and serum albumin tended to decrease from 4.1 to 4.0 ​g/dl (−0.1 ​g/dl). Compared with the changes in serum nutritional parameters during the 12 months after LSG, those during the subsequent 12 months of semaglutide treatment had greater reductions in serum albumin and vitamin B12.

**Fig. 3:**
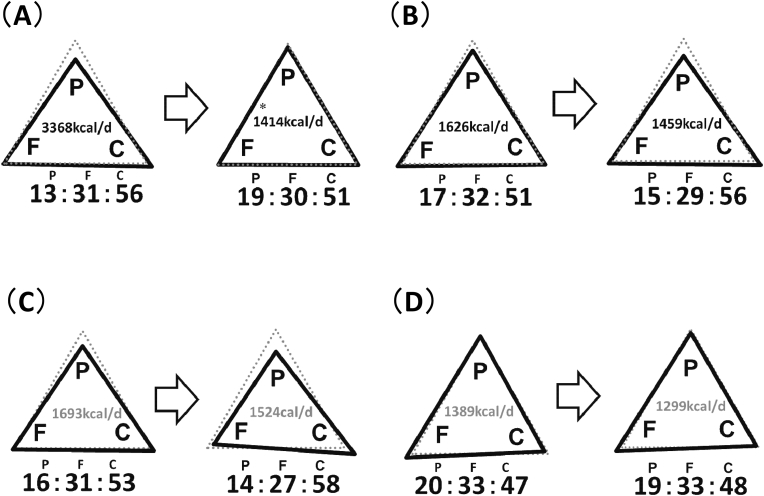
Comparison of changes in total energy intake and PFC ratio. (A) Baseline versus 12 months after LSG. (B) Before starting semaglutide versus after 12 months of once-weekly semaglutide treatment. (C) Patients with one meal per day replaced with a formula diet (FD) group. (D) Patients on conventional diet (CD) group. LSG, Laparoscopic sleeve gastrectomy; P, protein; F, fat; C, carbohydrate. ∗P ​< ​0.001 vs baseline.

Next, changes in intake of total energy, protein, fat, and carbohydrate during 12 months after LSG were analyzed (Fig. 3A and B). From before LSG to 12 months after LSG, total energy intake decreased significantly from 3368 to 1414 kcal/day, PFC ratio changed from 13:31:56 to 19:30:50, protein ratio increased (P < 0.0001) and carbohydrate ratio decreased significantly (P = 0.032). On the other hand, from before starting semaglutide to after 12 months of treatment, total energy intake decreased significantly from 1626 to 1459 kcal/day, PFC ratio changed from 17:32:51 to 15:29:56, protein ratio decreased (P = 0.038) and carbohydrate ratio increased significantly (P = 0.010).

Furthermore, there were no differences in changes in BW, BMI, HbA1c, metabolic parameters or measured nutritional metrics when comparing patients receiving initial GLP-1 analogue therapy with those switching from another GLP-1 analogue. In addition, there were no differences in the changes in these parameters according to the type or dose of the GLP-1 analogue used (data are not shown).

### 3.3 Effects of formula diet on measured nutritional metrics and body composition in patients receiving semaglutide after LSG

**Fig. 4:**
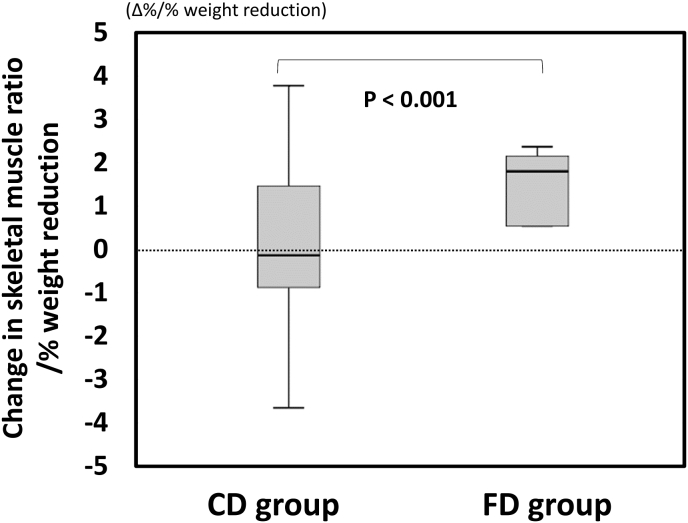
Comparison of changes in skeletal muscle ratio between formula diet (FD) and conventional diet (CD) groups after LSG and after 12 months of once-weekly semaglutide treatment. Comparison of the change per 1 % body weight change. Data are presented as median (interquartile range).

Patients who were treated with semaglutide after LSG were retrospectively divided into FD and CD groups to compare the changes in parameters from before starting semaglutide to after 12 months of treatment. For metabolic indicators, mean changes in fasting blood glucose in FD group and CD group were −37.5 mg/dl and −3.0 mg/dl, respectively; mean changes in HbA1c were −1.45 % and −0.40 % (data not shown). These indices were reduced significantly more in FD group than in CD group. There was no difference in the change in skeletal muscle ratio between FD and CD groups (data not shown), but when comparing the change per 1 % body weight change, the changes were 0.73 %/% (a median of 2.00 [−1.35-2.51] %/%) in FD group and −0.22 %/% (a median of −1.45 [−1.26-2.21] %/%) in CD group, with a significant difference between the two groups (P < 0.0001) (Fig. 4). As for the comparison of PFC ratios, they changed from 20:33:47 to 19:33:48in FD group and from 16:31:53 to 14:27:58 in CD group (Fig. 3C and D). In CD group, there was a trend of increase in carbohydrate ratio (P = 0.053).

## 4 Discussion

In this study, we examined the effect of once-weekly semaglutide on post-LSG weight and glycemic control, as well as on measured nutritional metrics and body composition in Japanese patients with type 2 diabetes treated with LSG for obesity and who showed weight recurrence and worsened glycemic control at more than 12 months after LSG. We also examined the effects of FD on these indices. Mean body weight, BMI, VFA, and HbA1c, which tended to increase at 12 months after LSG, decreased significantly after the patients were treated with once-weekly semaglutide for one year. Although metabolic surgery can achieve 30 % weight reduction and high remission rate of type 2 diabetes [22], studies have shown that some patients have relapse of type 2 diabetes due to postoperative weight recurrence [7,[23], [24], [25]], and some have poor metabolic improvement due to inadequate postoperative weight reduction [6,26]. The results of this study suggest that once-weekly semaglutide may be an effective treatment for weight recurrence and worsening diabetes after bariatric surgery, supporting the finding of a report [14]. In addition, semaglutide is known to increase insulin secretion in pancreatic β-cells [12]. In this study, CPR decreased after LSG, and it is interesting to note that once-weekly semaglutide treatment improved glycemic control while increasing CPR.

Semaglutide induces weight reduction through delayed gastric emptying and hypothalamic GLP-1 receptor-mediated appetite suppression [20]. On the other hand, semaglutide administration is often accompanied by gastrointestinal symptoms such as nausea, constipation, and gastroesophageal reflux disease [27]. Therefore, semaglutide administration may affect not only total energy intake, but also food preference and nutrient balance, and may adversely affect measured nutritional metrics and body composition. This study suggested that weight reduction by once-weekly semaglutide treatment was accompanied by significant decreases in serum albumin, vitamin B12 and zinc, as well as adverse effect on skeletal muscle, compared with the changes after LSG. In this study, total energy intake decreased markedly, protein ratio increased significantly, and carbohydrate ratio decreased significantly after LSG, whereas protein ratio was significantly reduced and carbohydrate ratio was significantly elevated after 12 months of semaglutide treatment. As dietary therapy for type 2 diabetes with obesity, a high-protein, low-carbohydrate diet has a positive effect on weight reduction and improves body composition and glycemic control, when total energy intake is restricted [28]. Weight reduction induced by GLP-1 analogs such as semaglutide is not solely attributed to body fat, but may also involve skeletal muscle [29]. In another study, treatment with GLP-1 analogs decreased appetite for and intake of high-fat foods such as meat, but did not decrease intake of low-fat, sweet foods such as fruits and cereals [20]. In this study, the protein ratio decreased significantly, and serum albumin, vitamin B12, and zinc decreased during semaglutide treatment, suggesting that the intake of meat, which is rich in these nutrients, may have decreased, resulting in lower animal protein intake and reduced skeletal muscle mass. Decreased skeletal muscle mass in patients with type 2 diabetes is a risk factor for poor glycemic control, decreased basal metabolic rate, and development of sarcopenia. Therefore, when prescribing once-weekly semaglutide, it is necessary also to manage nutrition to ensure that the diet is higher in protein and lower in carbohydrate.

FD is a high-protein, low-carbohydrate, low-fat food with adequate vitamins and minerals. A multicenter trial has shown that partial use of FD reduces body weight, VFA, HbA1c, triglycerides and systolic blood pressure, and increases HDL-C in patients with type 2 diabetes and obesity [16]. In this study, one notable finding was the significantly lower carbohydrate diet consumed in the FD group (PFC ratio: 20:33:47) compared to the CD group (16:31:53). Patients in the FD group receiving weekly semaglutide after LSG also had significantly lower HbA1c, fasting blood glucose, and improved skeletal muscle ratio, by maintaining a high-protein, low-carbohydrate diet. We propose partial use of FD as a new nutritional therapy that reinforces the weight reduction and glycemic control effects of semaglutide and reduces the nutritional disadvantages of semaglutide in patients with type 2 diabetes.

## 5 Limitations

There are several limitations in this study. This study had a retrospective, observational design and was a single-center experience. Only Japanese subjects were included in this study. It is possible that the criteria for the initiation of semaglutide after LSG were different in each case. The statistical significance of the analyses of nutrition data may be uncertain because of the difficulties to accurately calculate calorie and nutrient intake. The sample size, especially in FD group, was small and could be a significant obstacle in identifying a trend and significant relationship. The difference in the dose of semaglutide used in the patients could have led to a significant difference in the results. The possibility that other GLP-1 analogue prior to semaglutide administration may have influenced the response to semaglutide. A prospective study with a larger sample size is needed in the future.

## 6 Conclusion

● In Japanese patients with type 2 diabetes treated for obesity with LSG who showed weight recurrence after LSG, treatment with once-weekly semaglutide resulted in weight reduction and improved glycemic control.
● On the other hand, once-weekly semaglutide adversely affected their measured nutritional metrics with increased carbohydrate ratio, decreased protein ratio, decreases in some vitamins and minerals, and reduced skeletal muscle ratio.
● When patients using FD as partial meal replacement were treated with once-weekly semaglutide, they maintained a high-protein and low-carbohydrate diet and showed increased skeletal muscle ratio, although the small sample size in FD group may have led to a significant difference.

Partial use of FD may be a new nutritional therapy that reinforces the weight reduction and glycemic control effects of semaglutide and reduces the nutritional disadvantages of semaglutide in patients with type 2 diabetes.

## Author contribution (CRediT authorship contribution statement)

All authors made substantial contributions to conception and design, acquisition of data, or analysis and interpretation of data; took part in drafting the article or revising it critically for important intellectual content; gave final approval of the version to be published; and agree to be accountable for all aspects of the work.

## Ethical review

All procedures and data collection were conducted in accordance with the ethical standards of the institutional and Japanese national research committees or the ethical standards of the Helsinki Declaration of 1975. Opt-out informed consent protocol was used for this study. This study and consent procedure were reviewed and approved by the Ethics Committee of Toho University Sakura Medical Center, approval number S18061.

## Source of funding

No funding was received from agencies in the public, commercial or not-for-profit sectors.

## Declaration of AI and AI-assisted technologies in the writing process

No use of AI and AI-assisted technologies in this study.

## Declaration of competing interest

Atsuhito Saiki declares the following financial interests/personal relationships which may be considered as potential competing interests: a relationship with Novo Nordisk Pharma Co Ltd, Japan, that includes: speaking and lecture fees. The other authors declare that they have no known competing financial interests or personal relationships that could have appeared to influence the work reported in this paper. All authors have no financial, promotional, research or other relationship with the manufacturers of MICRODIET (Sunny Health Co. Ltd., Japan) or ObeCure (US Cure Co. Ltd., Japan).
